# Site-Specific and Time-Dependent Activation of the Endocannabinoid System after Transection of Long-Range Projections

**DOI:** 10.1371/journal.pone.0033537

**Published:** 2012-03-22

**Authors:** Sonja Kallendrusch, Constance Hobusch, Angela Ehrlich, Simone Ziebell, Natsuo Ueda, Gerd Geisslinger, Marco Koch, Faramarz Dehghani

**Affiliations:** 1 Institute for Anatomy, Leipzig University, Leipzig, Germany; 2 Lipid Signaling Forschungszentrum, Frankfurt, Germany; 3 Institute for Pharmacology, Goethe University, Frankfurt, Germany; 4 Department of Biochemistry, Kagawa University, School of Medicine, Kagawa, Japan; Centre national de la recherche scientifique, France

## Abstract

**Background:**

After focal neuronal injury the endocannabinioid system becomes activated and protects or harms neurons depending on cannabinoid derivates and receptor subtypes. Endocannabinoids (eCBs) play a central role in controlling local responses and influencing neural plasticity and survival. However, little is known about the functional relevance of eCBs in long-range projection damage as observed in stroke or spinal cord injury (SCI).

**Methods:**

In rat organotypic entorhino-hippocampal slice cultures (OHSC) as a relevant and suitable model for investigating projection fibers in the CNS we performed perforant pathway transection (PPT) and subsequently analyzed the spatial and temporal dynamics of eCB levels. This approach allows proper distinction of responses in originating neurons (entorhinal cortex), areas of deafferentiation/anterograde axonal degeneration (dentate gyrus) and putative changes in more distant but synaptically connected subfields (cornu ammonis (CA) 1 region).

**Results:**

Using LC-MS/MS, we measured a strong increase in arachidonoylethanolamide (AEA), oleoylethanolamide (OEA) and palmitoylethanolamide (PEA) levels in the denervation zone (dentate gyrus) 24 hours post lesion (hpl), whereas entorhinal cortex and CA1 region exhibited little if any changes. NAPE-PLD, responsible for biosynthesis of eCBs, was increased early, whereas FAAH, a catabolizing enzyme, was up-regulated 48hpl.

**Conclusion:**

Neuronal damage as assessed by transection of long-range projections apparently provides a strong time-dependent and area-confined signal for de novo synthesis of eCB, presumably to restrict neuronal damage. The present data underlines the importance of activation of the eCB system in CNS pathologies and identifies a novel site-specific intrinsic regulation of eCBs after long-range projection damage.

## Introduction

Functional deficits of the central nervous system (CNS) are provoked by direct or delayed tissue damage. Lesions of long-range projections as it occurs in spinal cord injury (SCI), traumatic brain injury (TBI) or stroke often results in severe neurological impairment finally leading to persistent clinical and social disabilities for the patients [1]. At cellular level, CNS injury provokes primary and secondary processes that involve biochemical cascades occurring from minutes to weeks [2]. Earlier experimental studies identified different regulatory, inflammatory or immunological factors that were closely associated with secondary damage such as lipid degradation, altered neurotransmitter release and receptor function [3], [4]. Neuronal cell death, reactive astrogliosis, microglia proliferation and activation are further consequences [5]. Research within the last two decades revealed that the endocannabinoid (eCB) system, among many different signaling pathways reflects a major modulating signaling machinery of excitotoxicity by influencing neuronal damage either in a destructive or protective way [6].The eCB system includes a diverse group of long-chain fatty acids, the eCBs, acting on two cloned cannabinoid receptors [7], namely the cannabinoid receptor type 1 (CB_1_) and type 2 (CB_2_) and several not yet cloned cannabinoid receptors [8], [9]. Well characterized eCBs like arachidonoylethanolamide (AEA) or 2-arachidonoylglycerol (2-AG) are partial or full agonists at CB_1_ and CB_2_ [10], [11]. However, some structurally-related fatty acids like oleoylethanolamide (OEA) and palmitoylethanolamide (PEA) both showing biological effects similar to endocannabinoids are considered as members of the eCB family even without binding to CB_1_ and CB_2_, respectively [12].

Chemically defined as N-acylethanolamines (NEA), AEA, OEA and PEA are synthesized on demand from membrane glycerophospholipids by highly specific enzymes. N-acyl phosphatidylethanolamine-selective phospholipase D (NAPE-PLD) reflects the rate limiting enzyme for biosynthesis of all three above mentioned NEA [13]. Generally, NEA have a short half-time and are selectively degraded. Fatty acid amide hydrolase (FAAH) is the best characterized enzyme that catabolizes NEA with the highest affinity for AEA [14], [15]. However, N-acylethanolamide-hydrolyzing acid amidase (NAAA) was recently discovered as a novel NAE-hydrolyzing enzyme with a preference for PEA [16].

The broad spectrum of eCB-mediated biological actions involves analgesia and anti-inflammation in the central nervous system as well as in peripheral tissues. However, very little is known about the time course of induction and precise regulation of the eCB system after transection of long-range projections in the origin and targeted brain areas. These long-range projections connecting developmentally distinct areas of the CNS are often harmed in various insults, like SCI, TBI and stroke. Organotypic entorhino-hippocampal slice cultures (OHSC) allow the investigation of such long-range projections since projection fibers (perforant pathway) originating from EC neurons terminate at the outer molecular layer of the DG and thus connects two evolutionary distinct brain regions. The deafferentiation of the dentate gyrus by perforant pathway transection is a powerful tool to study cellular and inflammatory responses not only at the lesion site but also on anterograde projection areas [17], [18], [19], [20], [21].

In the present study we thus assumed an involvement of the eCB system and a possible neuroprotective role of its members not only at origin areas but also on neuronal populations localized in distant regions. The regulation of AEA, OEA and PEA levels and their main synthesizing and catabolizing enzymes were studied up to 72 hours after PPT. Moreover, the respective cell type being responsible for eCB production, release and/or catabolism, namely neurons, microglia or astrocytes were determined.

## Methods

### Ethics statement

All animal experiments have been approved by the ethics committees of the German federal states of Hessen or Saxonia and were performed in accordance with the Policy on Ethics and the Policy on the Use of Animals in Neuroscience Research as approved by the European Communities Council Directive (89/609/EEC) amended by the directive 2010/63/EU of the European Parliament and of the Council of the European Union on the protection of animals used for scientific purposes.

### Organotypic Entorhino-Hippocampal Slice Cultures (OHSC)

OHSC were prepared from 8-day old Wistar rats. Animals were decapitated and the brains were dissected under aseptic conditions [20]. After removal of the frontal lobe and the cerebellum, the brains were placed in 4°C minimal essential medium (MEM; Gibco BRL Life Technologies, Eggenstein, Germany) containing 1% (v/v) glutamine (Gibco). A sliding vibratome (Leica VT 1000 5, Leica Microsystems AG, Wetzlar, Germany) was used to cut the brain horizontally in 350 µm-thick slices. The hippocampus was dissected and immediately placed on cell culture inserts (pore size 0.4 µm; Millipore, Schwalbach/Ts., Germany) and were cultured in 6-well culture dishes (Falcon, BD Biosciences Discovery Labware, Bedford, MA) containing 1 ml culture medium (50% (v/v) MEM, 25% (v/v) Hanks' balanced salt solution (HBSS, Gibco), 25% (v/v) normal horse serum (NHS, Gibco), 2% (v/v) glutamine, 1.2 mg/ml glucose (Braun, Melsungen, Germany), 0.1 mg/ml streptomycin (Sigma-Aldrich, Deisenhofen, Germany), 100 µg/ml penicillin (Sigma-Aldrich), 0.8 µg/ml ascorbic acid (Sigma-Aldrich) and 1 µg/ml insulin (Boehringer, Mannheim, Germany; pH 7.4)) per well. OHSC were cultured at 35°C in a fully-humidified atmosphere with 5% (v/v) CO_2_. The culture medium was changed every second day.

### Primary cell cultures

Primary cultures of hippocampal neurons were prepared using a modified method originally described by Brewer and colleagues [22]. Briefly, brains of P0 Wistar rats were removed and placed into a solution of ice-cold HBSS. The hippocampi were dissected and placed in Neurobasal Medium (Gibco) containing BSA (Sigma-Aldrich) and papain (1 mg/ml, Sigma-Aldrich) for 20 min at 37°C. Then neurons were isolated by tissue dissociation using Pasteur pipette, centrifuged for 10 min (45 g) and plated onto poly-L-lysine-coated coverslips. Cells were maintained in Neurobasal medium supplemented with B-27 (Gibco), GlutaMAX (Gibco) and penicillin/streptomycin at 37°C in a humidified atmosphere with 5% (v/v) CO_2_ for 2 weeks.

Primary microglial and astrocyte cell cultures were isolated from cerebral cortices of P1 neonatal Wistar rats. After removal of the meninges, cerebral cortices were dissociated in Ca^2+^/Mg^2+^-free HBSS, containing trypsin (4 mg/ml, Boehringer) and DNAse (0.5 mg/ml, Worthington, Bedford, MA, USA). Cells were plated into poly-L-lysine (Sigma-Aldrich) coated 75 cm^2^ tissue culture flasks (Falcon) containing DMEM (Gibco) supplemented with 4.5 g/l glucose (Gibco) and 10% (v/v) fetal bovine serum (FBS, Gibco), 1% (v/v) glutamine, 100 µg/ml penicillin and 100 µg/ml streptomycin.

Microglia was isolated from the astrocytic monolayer and incubated with control medium (DMEM supplemented with 2% (v/v) FBS, 1% (v/v) glutamine and 1% (v/v) penicillin/streptomycin). One day prior to all experiments, primary cultures of microglia and astrocytes were transferred into 24-well dishes coated with poly-L-lysine.

### Perforant Pathway Transection (PPT)

**Figure 1:**
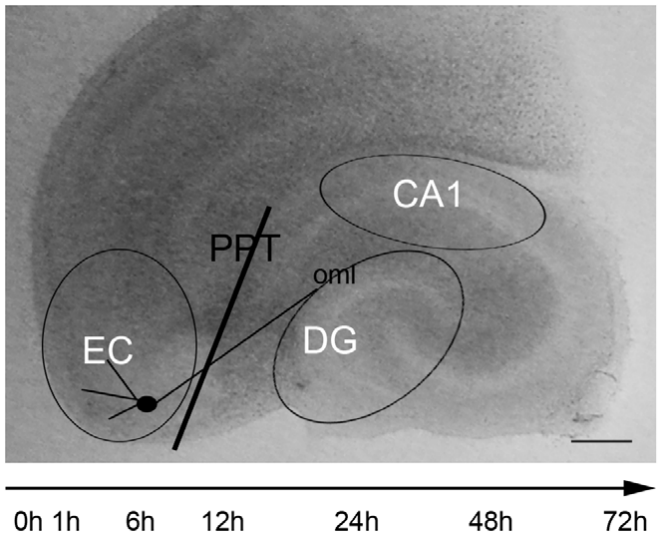
Overview of the perforant pathway transection (PPT) in OHSC. The black line indicates the transection of the axons originating from layers II and III of entorhinal cortex (EC) and projecting to the outer molecular layer (oml) of the dentate gyrus (DG). The areas of EC, DG and cornu ammonis (CA)1 were dissected as visualized by the open black cycles. OHSC were kept in culture for 6 days before PPT was set and tissue of the highlighted areas was collected 1 h, 6 h, 12 h, 24 h, 48 h and 72 h post lesion. Bar = 500 µm.

The PPT was mechanically set on day 6 *in vitro* (div) by use of a disposable ophthalmic scalpel equipped with a stainless steel blade (Feather, Osaka, Japan). Under a binocular (Zeiss, Jena, Germany), PPT was performed within OHSC through the perforant pathway following the sulcus between the hippocampus and the entorhinal cortex (EC) [23]. The EC, the dentate gyrus (DG) and the cornu ammonis 1 (CA1) region were dissected 0, 1, 6, 12, 24, 48 or 72 hours post lesion (hpl; Fig. 1).

### Neuronal damage

To analyze neuronal cell death, the medium of some OHSC was supplemented with 5 µg/ml propidium iodide (PI) 2 hours (h) prior to fixation with 4% (w/v) paraformaldehyde (Sigma-Aldrich) in 0.1 M phosphate buffer (PB). After fixation for at least 4 h, the cultures were washed twice with PB and embedded with Dako fluorescent mounting medium (Dako Diagnostika GmbH, Hamburg, Germany) and analyzed by a Zeiss LSM 510 Meta confocal laser scanning system (Zeiss, Göttingen, Germany). ImageJ software (U.S. National Institutes of Health, http://rsb.info.nih.gov/ij/download.html) was used for counting degenerating PI positive neuronal nuclei in the different EC, DG and CA1 regions.

### LC-MS/MS

Tissues of 3 OHSC were pooled and immediately shock frozen in liquid nitrogen and stored at −80°C. Homogenization was performed on ice to prevent degradation of eCBs or internal standards. The extraction of eCBs was conducted with a 9∶1 (v/v) ethylacetate/n-hexan solution [24]. The tissue was homogenized first in 70 µl ice cold H_2_O in a mixer mill 400 (Retsch, Haan, Germany) for 90 seconds at 25 Hz. As a next step, 20 µl were taken away from the homogenates to quantify the protein amount of beta-actin by Western Blot analysis (see below). 50 µl of the tissue samples were again homogenized together with 25 µl of internal standards, 50 µl ethylacetate/n-hexan and 50 µl H_2_O. After 3 minutes of centrifugation at 10,000 g, the organic phase was collected and the extraction procedure was repeated. The ethylacetate phases were evaporated under a gentle stream of nitrogen and assimilated in 25 µl acetonitril in glass vials. Finally, 10 µl were injected into the LC-MS/MS system (API, 5000, AB SCIEX, California, USA). For accuracy, quality standards were always extracted with the samples.

The reconstituted samples were analyzed for AEA, OEA and PEA. The respective deuterated AEA-d_8_, OEA-d_2_ and PEA-d_4_ were used as internal standards. HPLC analysis was performed under gradient conditions using a Luna HST C18 (2) column (100 mm L×2 mm ID, 2.5 µm particle size; Phenomenex, Aschaffenburg, Germany). MS and MS/MS analyses were performed on an API 5000 triple quadrupole mass spectrometer with a Turbo V source (Applied Biosystems, Darmstadt, Germany) in the negative ion mode. Precursor-to-product ion transitions of m/z 346→259 for AEA, m/z 354→86 for AEA-d_8_, m/z 324→86 for OEA, m/z 326→86 for OEA-d_2_, m/z 298→268 for PEA and m/z 302→258 for PEA-d_4_ were used for the multiple reaction monitoring (MRM) with a dwell time of 70 milliseconds. Concentrations of calibration standards, quality controls and unknowns were evaluated by Analyst software (version 1.4; Applied Biosystems). Variations in accuracy and intra-day and inter-day precision were <15% over the range of calibration. For each sample, endocannabinoid data was expressed in relation to the respective beta-actin level as obtained by Western Blot analysis (Image J 1.43, imagej.nih.gov/ij/download/) within the same sample. In each experiment protein extracts from controls and the corresponding PPT time matches were run onto the same gel to ensure similar conditions for both groups. Endocannabinoid values were then normalized and values obtained from controls were set as 100%. Changes after PPT were described in relation to their corresponding time controls.

### Western Blot analysis

For Western Blot analysis the tissue was collected and immediately stored in lysis buffer at −80°C. Protein extracts were obtained by sonication of tissues in lysis buffer containing 80 mM Tris, 70 mM SDS, 0,3 M Saccharose, 3 mM sodium orthovanadate and 0.5 mM phenylmethylsulfonyl fluoride (PMSF) at pH 7.4. Cell debris was removed by centrifugation for 10 min at 3000 g. Protein concentrations of the supernatants were determined by BCA test (Thermo Fisher Scientific, Rockford, USA). Equal protein amount of 5 µg were loaded onto a 12,5% (w/v) sodium dodecylsulfate–polyacrylamide gel. After gel electrophoresis, proteins were electrotransferred to nitrocellulose membranes. After blocking non-specific protein-binding sites for 1 h with 5% (w/v) milk (Carl Roth, Karlsruhe, Germany) or 5% (v/v) Roti-block solution (Carl Roth, Karlsruhe, Germany) in TBST, the membranes were incubated over-night with the respective following primary antibodies diluted in 5% (w/v) milk or 5% (v/v) Roti block in TBST: rabbit polyclonal antibody against human NAPE-PLD (diluted 1∶1000; cat.-No 10305 (aa 6–20), Cayman Chemicals, Ann Arbor, MI, USA), rabbit polyclonal antibody against human FAAH (diluted 1∶1000; cat.-No 101600 (aa 561–579), Cayman Chemicals), rabbit polyclonal antibody against rat NAAA (diluted 1∶5000; developed by N. Ueda, Kagawa, Japan) [25], guinea pig polyclonal antibody against murine CB_1_ (diluted 1∶2000; cat.-No CB1-G P-Af530-1, Frontier Science, Hokkaido, Japan), PPAR alpha (diluted 1∶1000; cat.-No PA1-822A, Thermo Scientific, Schwerte, Germany) and mouse monoclonal antibody directed against human beta-actin (diluted 1∶40,000; cat.-No A1978, Sigma-Aldrich). The next day, membranes were washed three times with TBST for 10 min and the secondary horse radish peroxidase-conjugated antibodies (anti-rabbit, 1∶1000; cat.-No PI 1000, Vektor laboratories, Burlingame, CA; anti-guinea pig 1∶1000;cat.-No P0141, DAKO Diagnostika GmbH, Hamburg, Germany or anti-mouse IgG, 1∶4000; cat.-No CP01, Millipore, Billerica, USA) were added for 1 h. Membranes were finally exposed to enhanced chemiluminescence (ECL detection system, Millipore) and the signal of bound antibody was visualized with radiographic films (Kodak, Stuttgart, Germany). Finally, semiquantification of the immunoreactive bands was performed with ImageJ image analysis software.

### Specificity tests for the antibodies used

The specificity of the antibodies was tested by preabsorption with the corresponding blocking peptides (NAPE-PLD, cat.-No 10303; FAAH, cat.-No 301600, Cayman Chemicals, respectively, NAAA, developed by Ueda's lab [25] and PPAR alpha, cat.-No PEP-025, Thermo Scientific). For preabsorption, each primary antibody was diluted in 5% (w/v) milk or 5% (v/v) Roti block in TBST and incubated with a five to ten-fold excess (by weight) of its blocking peptide for 1 h at room temperature with gentle shaking. Thereafter, the antibody-blocking peptide solution was applied to the Western Blot membranes and the subsequent procedures followed to the above described Western Blot protocol. The CB_1_ antibody was tested on CB_1_ (−/−) knock-out mice (kindly provided by Beat Lutz, Mainz, Germany, Fig. S1).

### Immunohistochemistry

The OHSC were fixed with a 4% (w/v) paraformaldehyde solution in 0.1 M PB for at least 4 h. After fixation, OHSC were washed in PB and incubated for at least 4 h in 15% (w/v) sucrose, followed by 30% (w/v) sucrose, respectively. Using a cryostat 3050 S (Leica, Wetzlar, Germany), the OHSC were sectioned (14 µm) and mounted on superfrost microscope slides (Thermo Scientific). To block unspecific binding sites sections were preincubated with normal goat serum for 30 min (1∶20 in PBS/Triton) followed by incubation with primary antibodies for 16 h in PBS/Triton containing 0.5% (w/v) bovine serum albumin: NAPE-PLD (diluted 1∶200), FAAH (diluted 1∶200), NAAA (diluted 1∶1000), CB_1_ (diluted 1∶100), PPAR alpha (diluted 1∶1000). Binding of the primary antibodies was visualized by means of the ABC method with a biotin-conjugated anti-rabbit IgG (diluted 1∶100; cat-No B7389 Sigma-Aldrich) as the secondary antibody, a horseradish peroxidase (HRP)-conjugated streptavidin complex (diluted 1∶100; cat.-No E2886 Sigma-Aldrich) and 3, 3-diamino-benzidine as the chromogen. For immunocytochemical characterization of different cell populations within OHSC, the following primary antibodies were used: mouse monoclonal antibody against NeuN as a neuronal marker (diluted 1∶200; cat.-No MAB377, Millipore), monoclonal mouse antibody against GFAP as a marker for astrocytes (diluted 1∶200; cat.-No 556330, BD Pharmingen) and biotinylated isolectin B_4_ (IB_4_) as a marker for microglial cells (diluted 1∶50; cat.-No FL-1201, Vector).

For triple-immunofluorescence staining, the primary antibodies against NAPE-PLD, FAAH, NAAA, CB_1_ or PPAR alpha, were combined with FITC-conjugated isolectin B_4_ and either with antibodies against NeuN or GFAP respectively. After incubation with primary antibodies for 16 h at room temperature, sections were washed three times with PBS followed by application of secondary antibodies Alexa fluor dye 568 (goat-anti rabbit IgG, 1∶500, cat.-No A-11011, Invitrogen) and Alexa fluor dye 633 (goat anti-mouse IgG, 1∶100, cat.-No A21050, Invitrogen, Karlsruhe, Germany) for 1 h. The preparations were finally coverslipped with Dako fluorescent mounting medium (Dako) and analyzed using a Zeiss LSM 510 Meta confocal laser scanning system.

### Statistical analysis

Data from at least three independent experiments were expressed as mean values (± standard error of the mean (SEM)). Data was statistically analyzed using ANOVA one way followed by Bonferroni posttests. Results with p<0.05 were considered as significant. Analysis was conducted with Graph Pad Prism software 5 (GraphPad software, La Jolla, USA).

## Results

### Perforant Pathway transection does not increase neuronal cell death in the EC, DG or CA1 area

The determination of neuronal cell death by counting the numbers of propidium iodide (PI) positive nuclei revealed very few PI positive nuclei in the EC (CTL: 0hpl, 0.0; 1hpl, 0.0, 6hpl, 0.0; 12hpl 0.0; 24hpl, 1.7; 48hpl, 1.7; 72hpl, 0.0; PPT: 0hpl, 0.0; 1hpl, 0.3, 6hpl, 0.0; 12hpl, 0.0; 24hpl, 1.3; 48hpl, 1.7; 72hpl, 0.0). Similar findings were observed in the DG (CTL: 0hpl, 0.0; 1hpl, 0.3, 6hpl, 0.3; 12hpl, 0.3; 24hpl, 0.7; 48hpl, 1.0; 72hpl, 0.0; PPT: 0hpl, 0.3; 1hpl, 0.7, 6hpl, 0.0; 12hpl, 1.7; 24hpl, 1.7; 48hpl, 1.3; 72hpl, 2.0) and CA1 region (CTL: 0hpl, 0.0; 1hpl, 0.0, 6hpl, 0.0; 12hpl, 0.0; 24hpl, 0.0; 48hpl, 0.7; 72hpl, 0.0; PPT: 0hpl, 0.0; 1hpl, 0.3, 6hpl, 0.0; 12hpl, 0.0; 24hpl, 0.3; 48hpl, 0.7; 72hpl, 1.0). There was no significant change in the number of PI positive nuclei in investigated regions (EC, DG, CA1) at all time-points assessed (p>0.05). No difference was found between controls and PPT slices (p>0.05).

### Endocannabinoid levels are regulated in a site specific and time dependent manner after PPT

**Figure 2:**
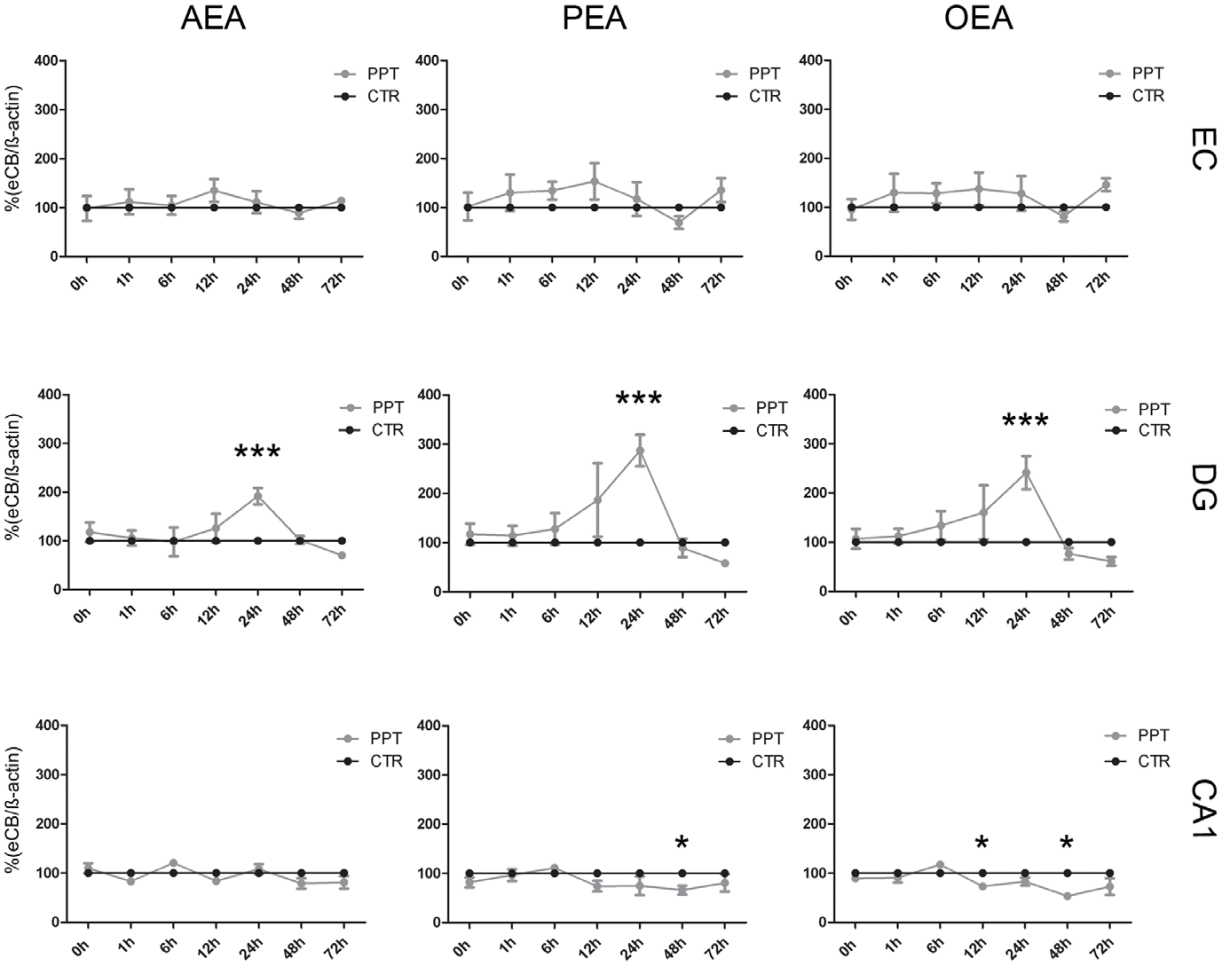
Endocannabinoid (eCB) levels in EC, DG and the CA1 region as normalized against ß-actin immunosignals. Control condition (CTR) of each time-point was set as 100% (black cycles). In EC AEA, PEA and OEA (gray circles, respectively) did not show any alteration in comparison to the respective CTR. In the DG, all investigated eCBs significantly increased 24hpl. In CA1, AEA did not differ from CTR after PPT. PEA showed a significant decrease 48hpl. OEA decreased at 12 and 48hpl. (n = 4, *p<0.05, ***p<0.001).

Axonal dissection/dendritic denervation as induced by PPT led to a site-specific intrinsic upregulation of AEA, PEA and OEA levels in the DG (Fig. 2). The eCB levels of non lesioned controls (CTR) were set to 100% and the PPT data was expressed in relation to their time controls, respectively. Under control conditions (0hpl) following mean values for NEA were found in the areas investigated (AEA: EC, 0.015 ng/ml; DG, 0.010 ng/ml; CA1, 0.018 ng/ml; PEA: EC, 0.828 ng/ml; DG, 0.769 ng/ml; CA1, 1.475 ng/ml; OEA: EC, 0.094 ng/ml; DG, 0.076 ng/ml; CA1, 0.150 ng/ml, respectively). In the EC region no significant changes in NEA levels were observed after PPT. AEA levels in the EC remained at control levels (0hpl, 115%; 1hpl, 139%; 6hpl, 74%; 12hpl, 121%;24hpl, 112%; 48hpl, 82%; 72hpl, 79%; p>0.05). Only at 1hpl a non significant elevation to 139% was observed (Fig. 2). At all time points investigated no significant change was detected for PEA (0hpl, 116%; 1hpl, 160%; 6hpl, 96%; 12hpl, 143%; 24hpl, 111%; 48hpl, 66%; 72hpl, 85%; p>0.05, Fig. 2A) or OEA (0hpl, 106%; 1hpl, 157%; 6hpl, 86%; 12hpl, 130%; 24hpl, 121%; 48hpl, 75%; 72hpl, 99%; p>0.05, Fig. 2).

In the DG AEA levels did not differ significantly from controls up to 12hpl (0hpl, 113%; 1hpl, 122%; 6hpl, 90%; 12hpl, 142%; p>0.05). At 24hpl mean AEA levels were significantly increased compared to controls (24hpl, 261%; p<0.001). Thereafter AEA levels declined (48hpl, 79%; p>0.05) and were below the respective time control at 72hpl (72hpl, 82%; p>0.05, Fig. 2B). PEA levels in the DG did not differ significantly from controls up to 6hpl (0hpl, 111%; 1hpl, 132%; 6hpl, 124%; p>0.05, Fig. 2). At 12hpl, the PEA level were elevated (12hpl, 193%; p<0.05) and reached the maximum at 24hpl (24hpl, 352%; p<0.001). PEA levels then declined (48hpl, 56%; p>0.05) and were reduced at 72hpl without reaching the significant threshold (72hpl, 76%; p>0.05). OEA levels after PPT in the DG were comparable to control levels up to 12hpl, (0hpl, 101%; 1hpl, 129%; 6hpl, 131%; 12hpl, 143%; p>0.05). At 24hpl OEA levels were maximal elevated (24hpl, 299%; p<0.001). Thereafter OEA levels declined and were at 48hpl and 72hpl lowered compared to the respective time controls (48hpl, 61%; 72hpl, 81%; p>0.05, Fig. 2).

In the CA1 region no significant changes were observed in AEA levels (0hpl, 114%; 1hpl, 107%; 6hpl, 113%; 12hpl, 66%; 24hpl, 90%; 48hpl, 59%; 72hpl, 106%; p>0.05, Fig. 2). PEA levels remained close to the control levels until 12hpl (0hpl, 92%; 1hpl, 119%; 6hpl, 111%; 12hpl, 76%; p>0.05, Fig. 2). At 24hpl (24hpl, 70%; p>0.05) PEA levels were below control levels reaching the significant threshold at 48hpl (48hpl, 38%; 72hpl, 92%; p<0.05). OEA levels in the CA1 region were comparable to the control levels up to 24hpl (0hpl, 84%; 1hpl, 112%; 6hpl, 132%; 12hpl, 77%; 24hpl, 64%; p<0.5). At 48hpl the OEA levels decreased significantly compared to the control levels (48hpl, 44%; p<0.05; 72hpl, 98%; p>0.5, Fig. 2).

### Differential regulation of enzymes and receptors of the eCB system after PPT

**Figure 3:**
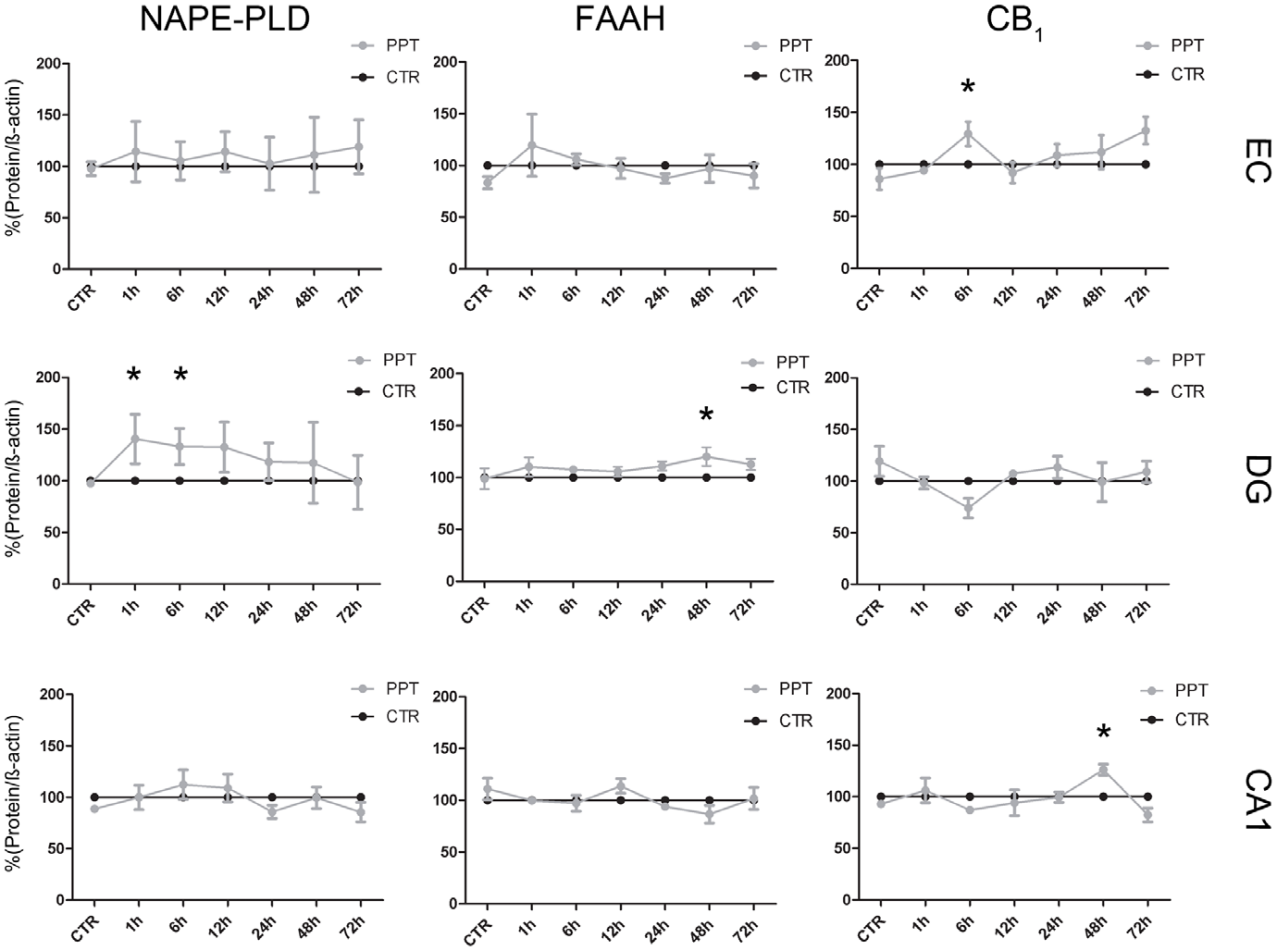
Relative amount of NAPE-PLD, FAAH and CB_1_ after PPT as compared to respective time control (CTR) that were set as 100%. Whereas NAPE-PLD and FAAH remained constant after PPT in EC, the amount of CB_1_ protein was significantly elevated 6hpl. In DG, NAPE-PLD was increased 1 and 6hpl. FAAH showed elevated levels 48hpl whereas CB_1_ did not show any alteration after PPT. In CA1, NAPE-PLD and FAAH remained unchanged after PPT. In contrast, CB_1_ levels were increased 48hpl. (n = 4, *p<0.05).

The site-specific and time-dependent changes in enzymes, namely NAPE-PLD, FAAH and NAAA as well as in receptors, namely CB_1_ and PPAR alpha were analyzed by Western blot. The specificity of the used antibodies was tested by means of preabsorption (Fig. S1A). The CB1 antibody showed no signal in CB_1_^−/−^ animals (Fig. S1B). For densitometric analysis the PPT data was expressed relative to their time-controls, respectively and the control levels were set as 100%. No change in NAPE-PLD was detectable after axonal damage in the EC (0hpl, 98%; 1hpl, 114%; 6hpl, 105%; 12hpl, 114%; 24h, 102%; 48hpl, 111%; 72hpl, 119%; p>0.05; Fig. 3). Neither FAAH (0hpl, 83%; 1hpl, 115%; 6hpl, 104%; 12hpl, 98%; 24h, 88%; 48hpl, 97%; 72hpl, 90%; p>0.05; Fig. 3) nor NAAA (0hpl, 88%; 1hpl, 89%; 6hpl, 79%; 12hpl, 98%; 24 h, 101%; 48hpl, 100%; 72hpl, 105%; p>0.05; Fig. S2) were significantly changed. After PPT CB_1_ protein (0hpl, 86%; 1hpl, 94%; 6hpl, 129%; 12hpl, 92%; 24 h, 101%; 48hpl, 108%; 72hpl, 132%; p>0.05; Fig. 3) was significantly increased after 1hpl (129%; p<0.05) and 72hpl (132%; p<0.05) in the EC only. PPAR alpha (0hpl, 83%; 1hpl, 105%; 6hpl, 93%; 12hpl, 112%; 24 h, 76%; 48hpl, 112%; 72hpl, 111%; p>0.05; Fig. S2) showed no alteration in PPT compared to the respective time control.

An increase of NAPE-PLD protein amount was observed in DG from 1hpl to 6hpl (0hpl, 97%; p>0.05; 1hpl, 140%; 6hpl, 133%; p<0.05; Fig. 3). Thereafter, NAPE-PLD protein decreased to control levels (12hpl, 133%; 24 h, 118%; 48hpl, 117%; 72hpl, 99%; p>0.05). The FAAH protein amount remained close to control levels up to 24hpl (1hpl, 110%; 6hpl, 108%; 12hpl, 106%; 24hpl, 111%; p>0.05). At 48hpl (120%; p<0.05) a significant peak of FAAH was observed that was no more visible at 72hpl (113%; p>0.05; Fig. 3). No change in the NAAA enzyme was detectable after dendritic denervation in the DG (1hpl 101%, 6hpl 98%, 12hpl 95%, 24 h 128%, 48hpl 105%, 72hpl 111%, Fig. S2). The protein amount of CB_1_ did not differ from the time control (0hpl, 119%; 1hpl, 101%; 6hpl, 77%; 12hpl, 107%; 24 h, 123%; 48hpl, 105%; 72hpl, 109%; p>0.05, Fig. 3). The PPAR alpha protein amount did not change in comparison to the time controls (1hpl 102%, 6hpl 107%, 12hpl 114%, 24 h 87%, 48hpl 107%, 72hpl 92%, p>0.05, Fig. S2).

In the CA1 region no alterations in NAPE-PLD were detected (0hpl, 89%; 1hpl, 100%; 6hpl, 112%; 12hpl, 109%; 24 h, 86%; 48hpl, 100%; 72hpl, 86%; p>0.05; Fig. 3). FAAH (0hpl, 111%; 1hpl, 100%; 6hpl, 97%; 12hpl, 114%; 24 h, 94%; 48hpl, 87%; 72hpl, 102%; p>0.05; Fig. 3) and NAAA (0hpl, 111%; 1hpl, 105%; 6hpl, 107%; 12hpl, 102%; 24 h, 99%; 48hpl, 106%; 72hpl, 81%; p>0.05; Fig. S2) remained unchanged after PPT. In PPT CB_1_ (0hpl, 93%; 1hpl, 106%; 6hpl, 87%; 12hpl, 94%; 24 h, 99%; 48hpl, 126%; 72hpl, 82%; p>0.05; Fig. 3) as well as PPAR alpha (0hpl, 86%; 1hpl, 97%; 6hpl, 135%; 12hpl, 110%; 24 h, 97%; 48hpl, 124%; 72hpl, 69%; p>0.05; Fig. S2) were not altered after PPT in CA1 as compared to the respective time controls.

### Distribution of NAPE-PLD, FAAH, NAAA, CB1 and PPAR alpha in OHSC

**Figure 4:**
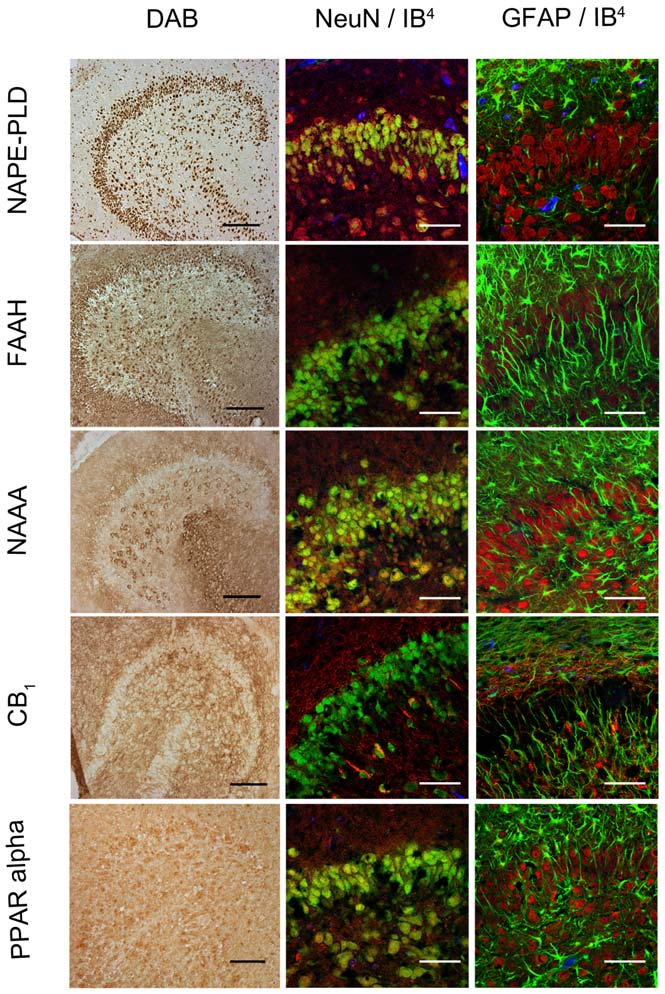
Immunocytochemical analyses of NAPE-PLD, FAAH, NAAA, CB_1_ and PPAR alpha in dentate gyrus 24hpl. Left column: DG in overview after DAB staining. Middle column; Triple staining of eCB system related proteins with NeuN and IB_4_. Right column; Triple staining of OHSC with eCB system related proteins in combination with NeuN and GFAP. The granular cell layer of the DG and interneurons in the hilus region showed a strong immunoreaction for NAPE-PLD. No overlap was observed with GFAP or IB_4_. The granular cell layer of the DG and especially interneurons in the hilus region were strongly immunoreactive for FAAH. No overlap was observed with GFAP or IB_4_. Please note the similarity of staining pattern of NAPE-PLD and FAAH immunoreactions. NAAA was found in perikarya and nuclei of neurons. In addition to granular cell layer of the DG and hilar neurons CA3 region was strongly labeled. Astrocytes and microglia seem free of NAAA. The entire molecular layer of the dentate gyrus showed a positive CB_1_ immunoreaction. CB_1_ was present in Microglia and to a lesser extent in astrocytes. PPAR alpha was observed in perikarya and nuclei of neurons. Microglia and Astrocytes showed a positive immunoreaction for PPAR alpha (n = 3, bars: left column = 100 µm, middle and right columns = 50 µm).

Localizations of NAPE-PLD, FAAH, NAAA, CB_1_ and PPAR alpha immunoreactions were investigated in situ by immunohistochemical staining with NeuN, GFAP and IB_4_ in sections obtained from OHSC at 24hpl. NAPE-PLD immunoreaction was found being co-localized with NeuN positive neurons and to a subset of IB_4_ positive microglia. GFAP positive astrocytes did not show a NAPE-PLD immunoreaction (Fig. 4). FAAH immunoreactions showed a robust cytoplasmic distribution in neuronal cells. Neither microglial cells nor GFAP positive astrocytes exhibited a detectable amount of FAAH (Fig. 4). Co-localization of NAAA immunoreaction with NeuN positive neurons were observed, mainly in the perikarya. In addition some nuclei showed positive immunoreactions for NAAA. There was no overlap of NAAA immunoreaction with IB_4_ or GFAP positive cells (Fig. 4). A strong CB_1_ immunosignal was detected in the inner and outer molecular layer of the DG. CB_1_ was present in some morphologically characterized inter-neurons of the hilus. Whereas IB_4_ positive microglia displayed a positive immunosignal for CB_1_ this reaction was weak when GFAP immunoreactive astrocytes were examined (Fig. 4). PPAR alpha was found in NeuN immunoreactive neurons, IB_4_ positve microglia and GFAP positive astrocytes. PPAR alpha was localized in vesicular structures close to the cell nucleus, apparently reflecting the Golgi apperatus throughout the entire OHSC.

### Cellular distribution of NAPE-PLD, FAAH, NAAA, CB_1_ and PPAR alpha in neurons, microglia and astrocytes

**Figure 5:**
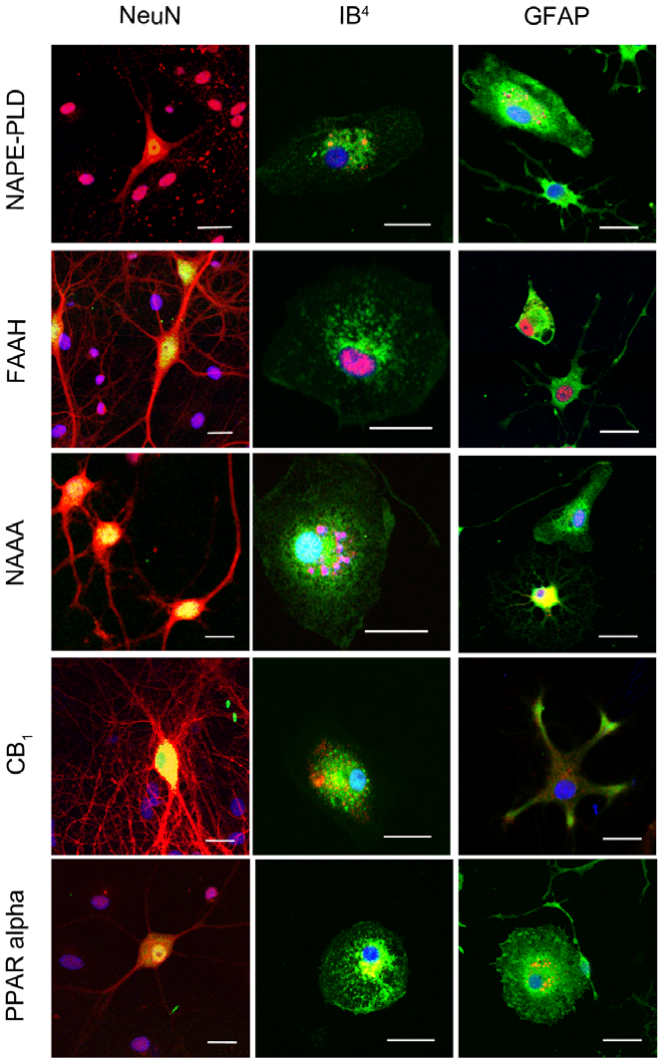
Immunocytochemical analyses of NAPE-PLD, FAAH, NAAA, CB_1_ and PPAR alpha in primary neuronal (left column), microglia (middle column) and astrocyte (left column) cell cultures. In neurons, NAPE-PLD was distributed in the perikarya but not in neuronal processes. In microglia NAPE-PLD immunoreactions was detectable in small vesicles closely located to the nucleus. NAPE-PLD was not found in protoplasmic astrocytes whereas fibrillary astrocytes showed an immunosignal comparable to microglia. The immunosignal for FAAH in primary neurons was localized in the cell bodies as well as in the primary branches of the neurites. In microglia and astrocytes, FAAH was localized within the nucleus, respectively. In fibrillary astrocytes a FAAH immunoreaction was additionally observed in vesicles. NAAA was clearly found in neurons but was barely found in microglia and astrocytes. In microglia NAAA showed a vesicular staining pattern close to the nucleus. Protoplasmic astrocytes were immunoreactive for NAAA whereas fibrillary astrocytes did not show any NAAA immunosignal. A robust CB_1_ immunoreaction was found in neurons. To a lower extent, CB_1_ immunosignals were detectable in microglia and astrocytes. Primary neurons showed a strong PPAR alpha immunoreaction in their Perikarya and nuclei. Microglia and astrocytes were positive for PPAR alpha immunoreaction in a vesicular pattern. (n = 3, bar = 20 µm).

In isolated primary neuronal cell cultures NAPE-PLD immunoreaction was observed within neuronal perikarya and processes. Similar to OHSC, NAPE-PLD was found close to the nuclei in microglial cells. In primary astrocyte culture fibrillary astrocytes displayed a weak, protoplasmic astrocytes a robust NAPE-PLD immunoreaction (Fig. 5). Primary neurons, microglia and astrocytes showed a FAAH immunoreaction that was localized close to the nuclei, respectively (Fig. 5). An additional nuclear labeling was observed in microglia and astrocytes. Isolated primary neurons showed a NAAA immunoreaction that was found distributed in the perikarya and the secondary branches. Microglia was also labeled with the NAAA antibody. Fibrillary astrocytes displayed NAAA immunoreactions within their cell bodies and to a minor extent in their processes (Fig. 5). Neurons showed a strong CB_1_ immunoreaction in perikarya, primary and secondary neuronal processes and spines. Microglia and astrocytes demonstrated a CB_1_ immunoreation, although the immunoreaction in astrocytes was very weak (Fig. 5). PPAR alpha immunoreaction was detectable in primary neurons, microglia and astrocytes (Fig. 5). In neurons PPAR alpha was scattered over the entire perikarya, whereas in glial cells it was found close to the nuclei, respectively.

## Discussion

Widespread secondary neurodegeneration occurs in the CNS after traumatic brain injury (TBI), spinal cord injury (SCI) and other CNS pathologies involving lesion of long-range projections. Recent studies demonstrated the neuroprotective properties of eCBs in various models of neuronal lesion in vitro and in vivo. It is well accepted that the eCB system represents a major modulator of synaptic transmission e.g. the ability of eCB to counteract excitotoxicity by reducing the calcium influx in excitatory glutamatergic neurons [26], [27]. In previous studies we found a strong variation between different cannabinoids with regard to their efficacy to prevent excitotoxic neuronal injury and to their mode of action on neuroprotection. Whereas AEA and the phytocannabinoid THC failed to protect dentate gyrus neurons, PEA as well as the endocannabinoids NADA and 2-AG and the synthetically designed cannabinoid WIN 55–212, 2 clearly showed neuroprotective effects. While CB_1_ receptor activation was responsible for WIN 55–212, 2 and NADA-mediated neuroprotection, PEA mediated its neuroprotective effects via dual PPAR alpha activation on microglial cells and neurons. Finally 2-AG was shown to activate abn-CBD receptors on microglial cells to mediate its neuroprotective effects [27], [28], [29], [30], [31].

Since some modes of action of eCB during detrimental processes in the brain are well understood, very little is known about the intrinsic regulation of the eCB system after transection of long-range projections in origin and target areas. Thus, by using the PPT model in OHSC the present study has been designed to investigate the intrinsic regulation of eCBs after lesion of long-range projections. This model, well known and highly accepted, allows a clear differentiation between anterograde, retrograde and more distant area changes induced by axonal injury. It should be considered that slice cultures derived from young animals show spontaneous seizure like events when investigated shortly after dissection [32] but these events disappear when cultures are kept in vitro for 7–21 days [33], [34], [35]. The activation of endocannabinoid system during the course of status epilepticus has very recently been reported in humans [36]. In the present investigation we didn't have noticed elevated endocannabinoid values under control conditions. Furthermore, the PPT group has always been analyzed in comparison to time match controls suggesting that changes in the amount of eCBS are most likely due to perforant pathway transection rather than an increase by spontaneous seizure like events.

The combination of the PPT model with the precise dissection of differentially linked regions forms a highly convenient model allowing profound insights in intrinsic regulation of eCB system after long-range projection damage.

We first examined neuronal cell death after PPT in OHSC and found that after 1, 6, 12, 24, 48, and 72hpl no significant changes in the number of PI^+^ neuronal nuclei was observed when compared to unlesioned control OHSC. These findings are in accordance with previous reports showing almost no neurodegeneration after PPT in dentate gyrus [19]. We then precisely dissected the specific areas, namely entorhinal cortex (EC), dentate gyrus (DG) and cornu ammonis 1 region (CA1) in controls and after PPT and investigated the regulation of AEA, PEA and OEA.

Small but not significant changes were observed in the EC, whereas a significant and robust increase in the concentration of all three eCBs investigated was found 24hpl in the DG. In the CA1 region a decrease of PEA and OEA but not AEA was seen after 12 and 48hpl. These findings indicate a spatial and temporal regulation of NEA mostly affecting the target area of long-range projections. Little is known about the specific regulation of NEA after transecion of long-range projections. In several reports enhanced AEA levels have been detected after various pathological events indicating a compensatory mechanism to brain damage [37], [38], [39]. In human studies, elevated AEA levels were measured in the cerebrospinal fluids of patients with Parkinson disease or after stroke and the increase in AEA was directly associated with brain damage [40], [41]. With regard to long-range projections, our data now asks for an additional consideration of more distant areas than the PPT, e.g. denervated spinal cord neurons as a source for AEA production to concern the regulation of eCB after transaction of long-range somato-afferent and/or efferent projections.

As a known CB_1_ agonist AEA activates at cellular level mitogen-activated protein kinase phosphatase-1 (MKP-1) decreasing MAPK signal transduction in microglial cells abolishing NO release and protects against over-activation of microglia [42], [43]. Therefore, the increase of AEA levels 24hpl in our study might reduce the microglia activation leading to neuroprotective effects. Stella and his group found in BV-2 cells an increase in microglia motility by AEA [44]. Similar to AEA, increased PEA levels observed here at 24hpl were also measured in inflamed tissues undergoing severe cell damaging processes, ischemic conditions and glutamate induced neurotoxicity and demonstrated neuroprotective properties [27], [45], [46]. One underlying mechanism seems an enhanced action of AEA on the vanilloid receptor caused by increased PEA levels, the so called entourage effect [47]. Further, PEA is reported to protect neurons via activation of PPAR alpha in neurons, microglia [27] and astrocytes [48]. OEA is suggested as a modulator of satiety, inflammation, lipid metabolism and antinociceptive effects, due to its activity at receptors such as TRPV1, GPR119 or PPAR gamma [46], [49], [50], [51]. The increase of OEA at 24hpl in the present study go along with a study of Galan-Rotriguez, demonstrating neuroprotective effects of OEA in dopamine neurons in the substantia nigra after 6-OHDA treatment [52]. Enhanced OEA levels were found to reduce microglia activation [53], [54]. Inhibition of reactive microglia by OEA might explain the lack of neurodegeneration after PPT in our system.

The diverse regulation of NEA in different brain regions after PPT demonstrates a specific involvement of eCBs in distinct damage regulation in the brain. Whether NEA perform different tasks or are separately regulated or act orchestral after PPT needs further investigation. Hansen et al. mentioned the difficulty of PEA and OEA data interpretation due to their structural similarity and comparable regulation to anandamide [55].

As a next step we investigated the time-dependent and spatial regulation of enzymes responsible for synthesis and hydrolysis of the NEAs. NAPE-PLD, synthesizing NEA from NAPE [13], [56], was elevated early between 1hpl and 6hpl after PPT but decreased to control levels at 72hpl. This time course of NAPE-PLD might explain the NEA elevation. A recent study showed a tight relation between regulation of NAPE-PLD and PEA levels and its impact on cell functions. It was shown that LPS decreased NAPE-PLD protein amount in macrophage cell cultures, leading to decreased PEA levels which finally led to subsequently exacerbated inflammatory reactions [57]. Interestingly, mice lacking NAPE-PLD did not exhibit lowered PEA levels [57]. It was assumed that two further synthesizing pathways compensate the lack of NAPE-PLD and take over NEA synthesis: (1) phospholipase C (PLC) creates a phosphor-NEA that is further catabolized by a phosphatase to NEA or, (2) via a combined action of a phospholipase B (Abh4) and a phosphodiesterase (GDE1) that hydrolyzes glycerophospho-NAE (GP-NAE) [58], [59]. It remained unclear whether these additional pathways are functionally activated in the presence of NAPE-PLD or were compensatory upregulated as well whether these alternative pathways are also involved in NEA synthesis after PPT.

Whereas FAAH was found being predominantly localized in endoplasmic reticulum [14] and able to hydrolyze all three here investigated acylethanolamides [16], NAE-hydrolyzing acid amidase (NAAA) detected in lysosomes [60] is responsible for the hydrolysis of acylethanolamides of less than 18 carbon atoms, a group of NEAs to which PEA significantly belongs to. FAAH was up-regulated at 48hpl, whereas NAAA protein remained unchanged at any time points after PPT. The up-regulation of FAAH was supposed to be responsible for the down-regulation of the NEA found at 48hpl. Evidence exists that reduction in NEA level by FAAH correlated with neurotoxic properties, as the FAAH inhibitor MAFP reduced apoptosis in the neocortex [61] or the inhibitor URB 597 decreased the infarct volume when administered before the focal cerebral ischemia [62]. Also other FAAH inhibitors were found to be beneficial for the outcome of neurological diseases [63]. Further investigations are required to figure out whether the here observed endogenous up-regulation of FAAH display the natural end of the inflammatory reaction or is directly involved in protective/harming effects.

Though, the enzymatic machinery for biosynthesis and catabolism of eCB is strongly affected by PPT in DG and CA1, we only found a variation for a potential target receptor of AEA, namely CB_1_ in the EC. This is in line with findings showing that middle cerebral artery occlusion in rats enhanced CB_1_ expression from 2 h on that persisted for 72 h in the ischemic region only [64]. The effects of ischemic insults upon CB_1_, however, is unclear and seems to depend from ischemic condition, time and species investigated [63]. Studies of differential regulated CB_1_ receptors on glutamatergic and GABAergic synapses might clarify these coherences [38]. A nuclear receptor for NEA is PPAR alpha [49], [65]. Application of exogenous PEA was reported to enhance PPAR alpha activation. Furthermore, selective NAAA inhibitors were reported to up-regulate the endogenous PEA levels and subsequently increase PPAR alpha activity [66], [67].

To clarify the protein regulation responsible for the observed NEA levels, cellular distribution of NAPE-PLD, FAAH, NAAA, CB_1_ and PPAR alpha were investigated by immunohistochemistry. A clear neuronal distribution in primary neurons as well as in OHSC was observed for NAPE-PLD and corresponded to previously described results [68]. NAPE-PLD was found in primary cultures of both microglia and astrocytes, however only microglia was immunoreactive in OHSC. In strongly inflamed postmortem brain specimens of MS patients NAPE-PLD immunoreactive microglia and astrocytes were observed [69]. FAAH clearly showed a neuronal distribution as previously reported by Cravatt et al. [14]. In OHSC FAAH was not seen in microglia neither in PPT nor in controls whereas primary microglia cell cultures expressed FAAH as shown in literature [70], [71]. In accordance to previous reports in Alzheimer disease we found in primary cell cultures, low FAAH expression in fibrillary and high FAAH expression in protoplasmic astrocytes [72]. To our knowledge, we showed here for the first time a clear neuronal NAAA distribution. NAAA was absent in IB_4_ and in GFAP positive cells in OHSC but was surprisingly present in primary cell cultures. So far, NAAA was solely described in macrophages as a lysosome associated protein [16]. Moreover, fibrillary astrocytes also showed high NAAA expression whereas protoplasmic astrocytes displayed weak NAAA in their nuclei only. In general the discrepancies between the findings especially for microglial cells regarding NAPE-PLD, FAAH and NAAA expression in primary cell cultures and complex OHSC might be due to the activation state of microglia in isolated cell cultures and the lack of factors present in CNS milieu. No obvious change over time was observed in histological staining with CB_1_. The potency of glial cells to synthesize and catabolize NEA under physiological or pathological conditions represents the high flexibility and complexity of the eCB system. Primary cultures displayed a different picture of enzyme and receptor distribution as in the complex model of OHSC. We therefore can state that in OHSC neurons and microglia produce NEA whereas hydrolysis of NEAs mainly happens in neurons. Depending on the lesion paradigm the machinery for production and degradation of NEA can be up-regulated in astrocytes as well.

Taken together the activation of the eCB system after neuronal damage by transection of long-range projections apparently provides a strong time-dependent and area confined signal for de novo synthesis of eCB, presumably to prevent or restrict neuronal damage. The present data underlines the importance of eCB in CNS pathologies and identified a site-specific intrinsic regulation of eCB levels in long-range projection damage. The highly dynamic eCB system represents an intrinsic, presumably protective system in the CNS.

## Supporting Information

Figure S1 **Specificity test for antibodies.** A: Specificity test for antibodies against NAPE-PLD, FAAH, NAAA and PPAR alpha by Western blot analyses. The antibody against NAPE-PLD showed two immunoreactive bands of about 46 kDa (1). Both bands were blocked by use of the respective blocking peptide (2). Similar to NAPE-PLD immumoreactive bands for FAAH, NAAA and PPAR alpha disappeared after preabsorption with respective peptides. B: NAPE-PLD, FAAH and NAAA fluorescent staining after preincubation of sections with respective blocking peptides or CB_1_ fluorescent staining in sections derived from CB_1_ knock out animals (Bar = 50 µm).
(TIF)

Figure S2 **Time-dependent regulation of NAAA and PPAR alpha in OHSC.** The data was shown in relation to the matching time controls that were set as 100%. In all regions investigated (EC, DG and CA1) no significant difference was found in OHSC after PPT as compared to controls (CTR).
(TIF)
