# Supplementary figures and images for: Site-Specific and Time-Dependent Activation of the Endocannabinoid System after Transection of Long-Range Projections
Source: PLoS One. 2012 Mar 22;7(3):e33537. doi: 10.1371/journal.pone.0033537 (PMC3310878; doi:10.1371/journal.pone.0033537)

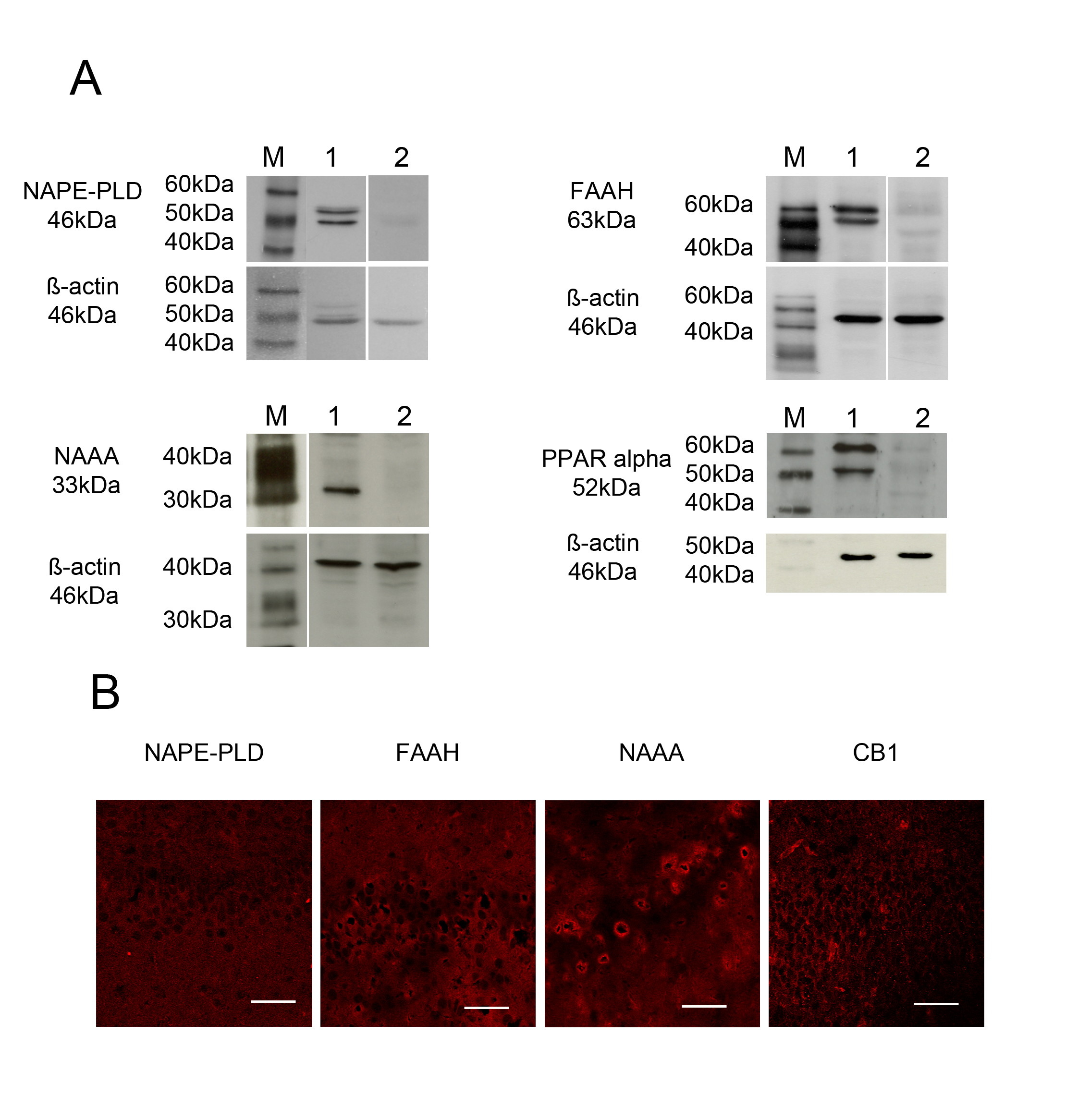

Supplement: Figure S1 — Specificity test for antibodies. A: Specificity test for antibodies against NAPE-PLD, FAAH, NAAA and PPAR alpha by Western blot analyses. The antibody against NAPE-PLD showed two immunoreactive bands of about 46 kDa (1). Both bands were blocked by use of the respective blocking peptide (2). Similar to NAPE-PLD immumoreactive bands for FAAH, NAAA and PPAR alpha disappeared after preabsorption with respective peptides. B: NAPE-PLD, FAAH and NAAA fluorescent staining after preincubation of sections with respective blocking peptides or CB1 fluorescent staining in sections derived from CB1 knock out animals (Bar = 50 µm). (TIF) [file pone.0033537.s001.tif]

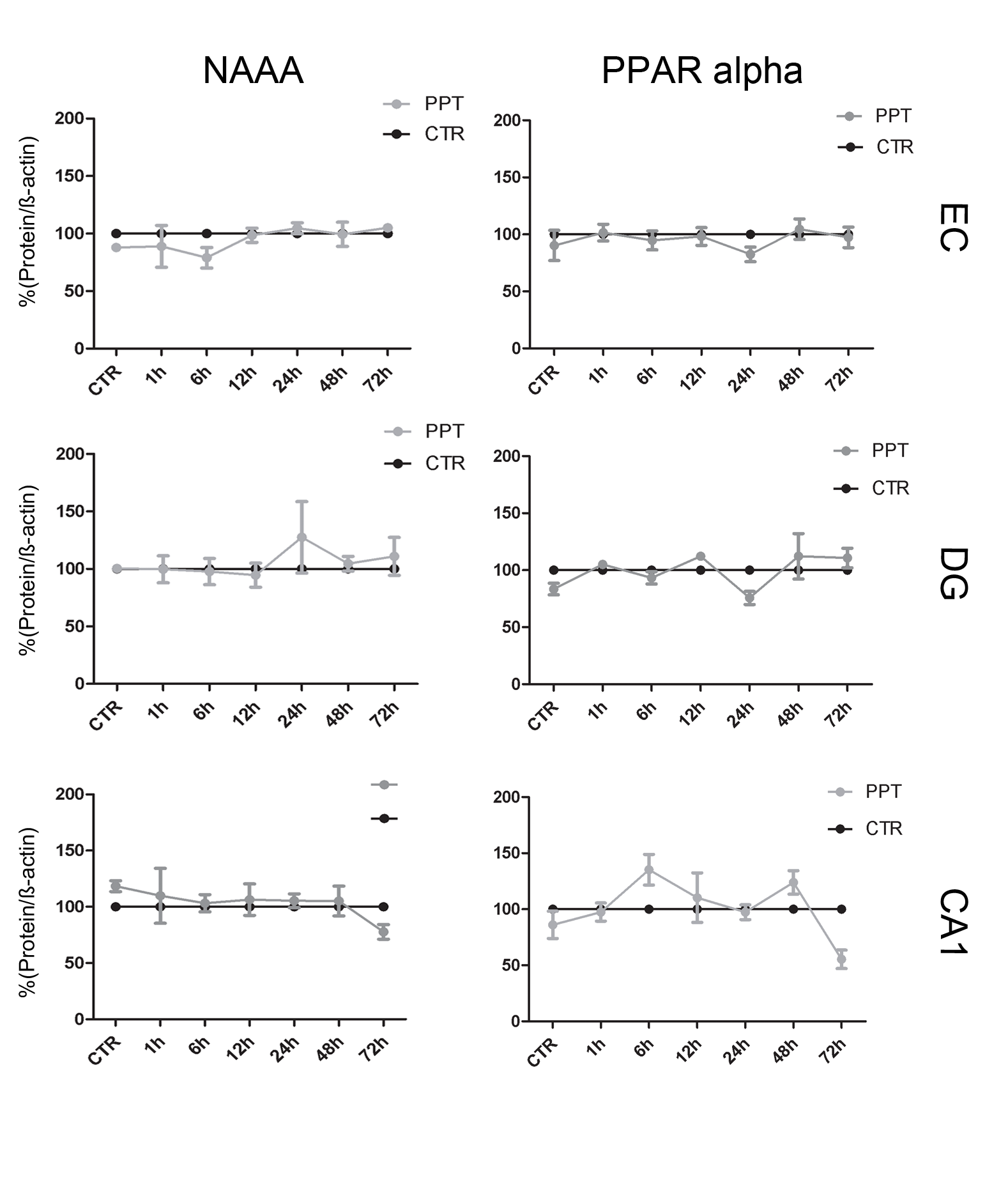

Supplement: Figure S2 — Time-dependent regulation of NAAA and PPAR alpha in OHSC. The data was shown in relation to the matching time controls that were set as 100%. In all regions investigated (EC, DG and CA1) no significant difference was found in OHSC after PPT as compared to controls (CTR). (TIF) [file pone.0033537.s002.tif]
